# Supplementary material for: Comparative Study of Short-Term Efficacy and Safety of Radical Surgery with or without Hyperthermic Intraperitoneal Chemotherapy in Colorectal Cancer with T4 Stage: A Propensity Score Matching Analysis
Source: J Clin Med. 2023 Feb 1;12(3):1145. doi: 10.3390/jcm12031145 (PMC9917540; doi:10.3390/jcm12031145)
Supplement: Supplementary file 1 [file jcm-12-01145-s001.zip › jcm-2134670-supplementary.pdf]

Supplementary Table S1. Definition of CTCAE V5.0 adverse events

Adverse event	Definition	I	II	III	IV
Anemia	A disease characterized by a decrease in the total amount of hemoglobin in 100 ml of blood	Hemoglobin < lower limit of normal value ~ 10.0 g / dl<Lower limit of normal value ~ 6.2mmol/l<Lower limit of normal value ~ 100g / L	Hemoglobin < 10.0 ~ 8.0g/dl<6.2 ～4.9mmol/L；< 100～80g/L	Hemoglobin < 8.0g/dl<4.9mmol/L；< 80 g/L；Blood transfusion is needed	Life threatening; Urgent treatment is needed
Hypoalbuminemia	The results of laboratory examination showed that the concentration of albumin in blood was low	< lower limit of normal value ~ 3 g / dl<Lower limit of normal value ~ 30 g / L	<3～2 g/dL；<30～20 g/L	<2 g/dL；<20 g/L	Life threatening; Urgent treatment is needed
Myelosuppression	A disease characterized by decreased hematopoietic function of bone marrow	Slightly too few cells or a decrease of ≤ 25% compared with the total number of normal cells in this age group	Moderately too few cells or a decrease of > 25% and < 50% compared with the total number of normal cells in this age group	Severe cytopenia or reduction of > 50% and ≤ 75% compared with the total number of normal cells in this age group	The aplastic disorder lasted for more than 2 weeks
Wound complications	New abnormalities in the existing wound site	It can be observed that local treatment is needed	Local care is required	Surgery is needed	Life threatening; Urgent treatment is needed

Abdominal infection	Abdominal infection	-	Oral administration is required (e.g. antibiotics, antifungal or antiviral drugs)	Intravenous antibiotics, antifungal or antiviral drugs are needed; Invasive treatment is needed	Life threatening; Urgent treatment is needed
Pulmonary infection	Infectious diseases, including pneumonia, occurring in the lungs.	-	Moderate symptoms; Oral medication (antibiotics, antifungal or antiviral drugs) is required	Intravenous antibiotics, antifungal or antiviral drugs are needed; Invasive treatment is needed	Life threatening; Urgent treatment is needed
Postoperative bleeding	Postoperative disorder characterized by bleeding	The symptoms are mild and need no treatment	Moderate bleeding requiring blood transfusion (blood transfusion volume < 2 units (infants < 10cc / kg))	Blood transfusion volume > 2 units (infants > 10 cc / kg), invasive treatment and hospitalization are required	Life threatening; Urgent treatment is needed
Anastomotic leakage	Rupture of gastrointestinal anastomosis and leakage of contents	The diagnostic findings were asymptomatic; No treatment required	Symptomatic; Need treatment	Serious; Invasive treatment is needed	Life threatening; Urgent treatment is needed
Ileus	A disease characterized by the inability of the gut to transport intestinal contents	No clinical symptoms, only radiation observation is required	Symptomatic; Gastrointestinal function changes; fasting	Severe changes of gastrointestinal function; Total parenteral nutrition is required; Catheterization is needed	Life threatening; Urgent treatment is needed

Supplementary Table S2. HIPEC drug utilization

Drug	No. (%)	Dosage (mg/m2)
Raltitrexed	44 (26.7)	2
Lobaplatin	99 (60.0)	35
Mitomycin	16 (9.7)	35
Oxaliplatin	6 (3.6)	35

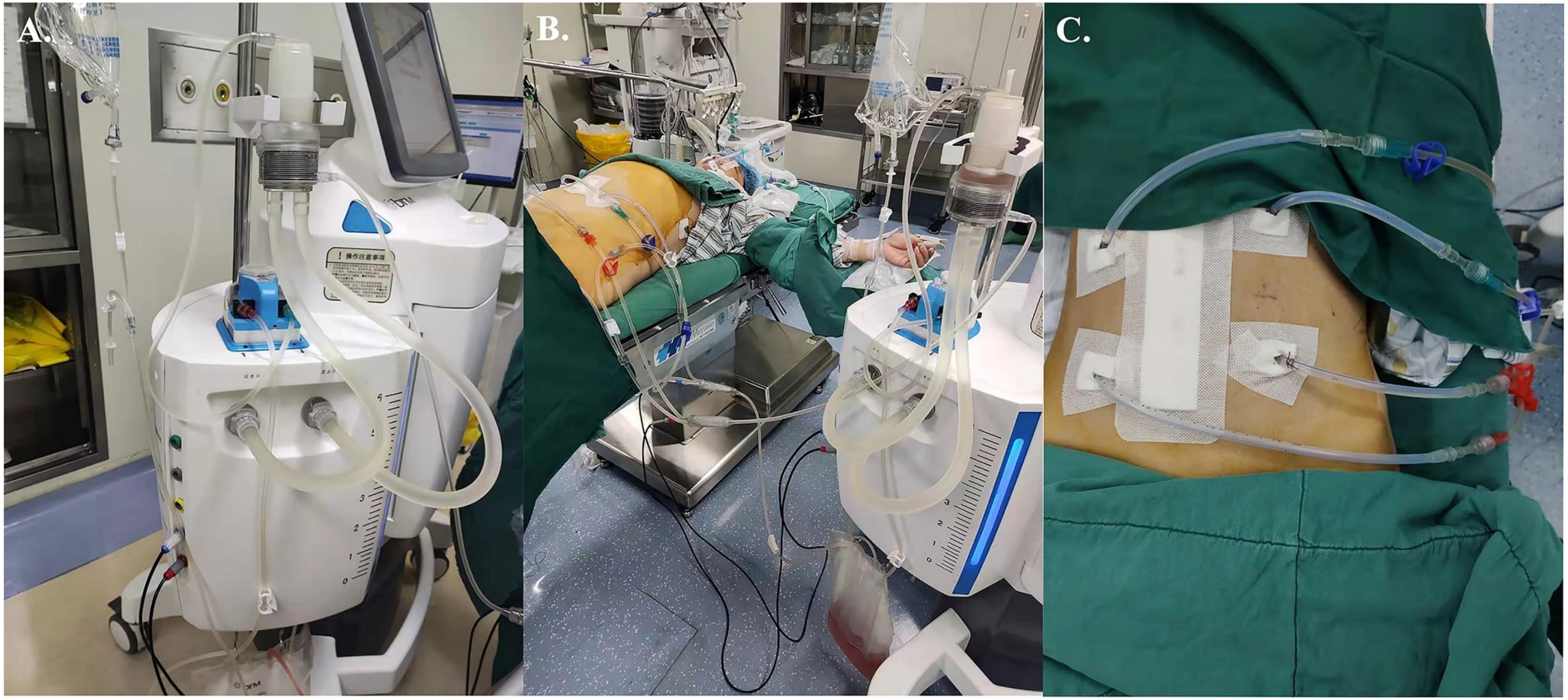

Supplementary Figure S1. Perfusion equipment and pipe connection. (A)The BRTRG-I hyperthermic perfusion intraperitoneal treatment system (Bright Medical Tech, Guangzhou, China). (B)Pipe connection. The red tube is the inlet tube, and the blue tube is the outlet tube. The perfusion fluid enters the abdominal cavity from the red tube below and flows out through the blue tube above. (C)Four tubes were used for circulatory perfusion; two each were placed in the upper and lower abdomen.

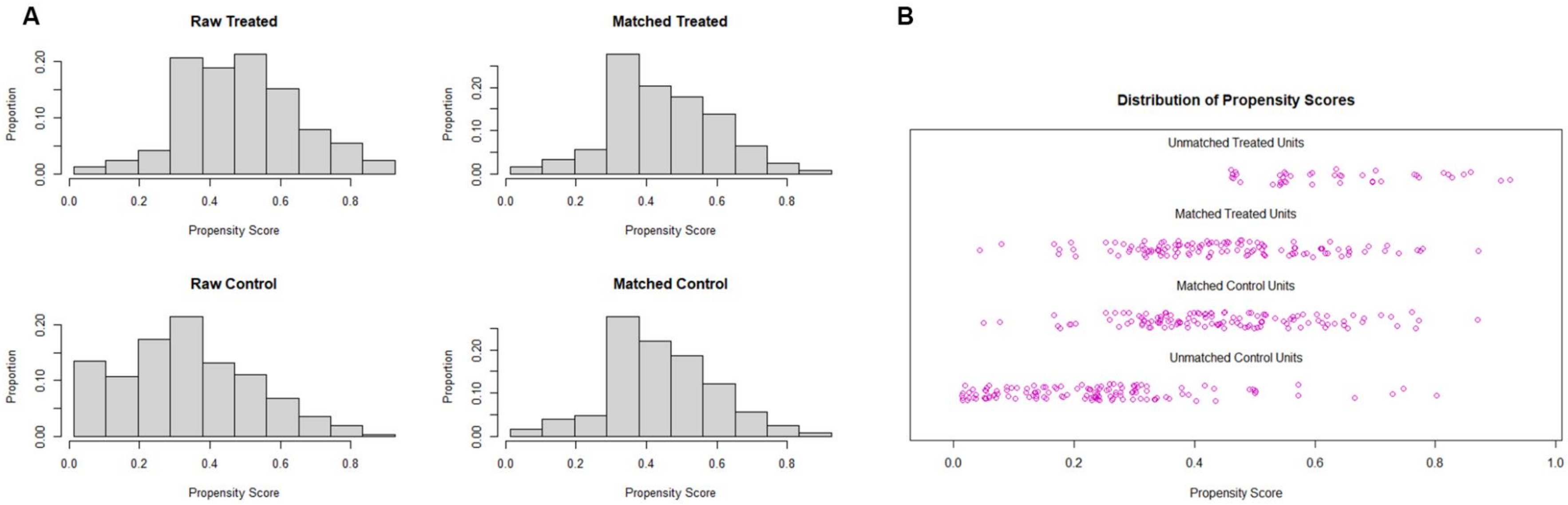

Supplementary Figure S2. Propensity score matching (PSM). (A) Histogram of propensity score. (B) Distribution of propensity score.

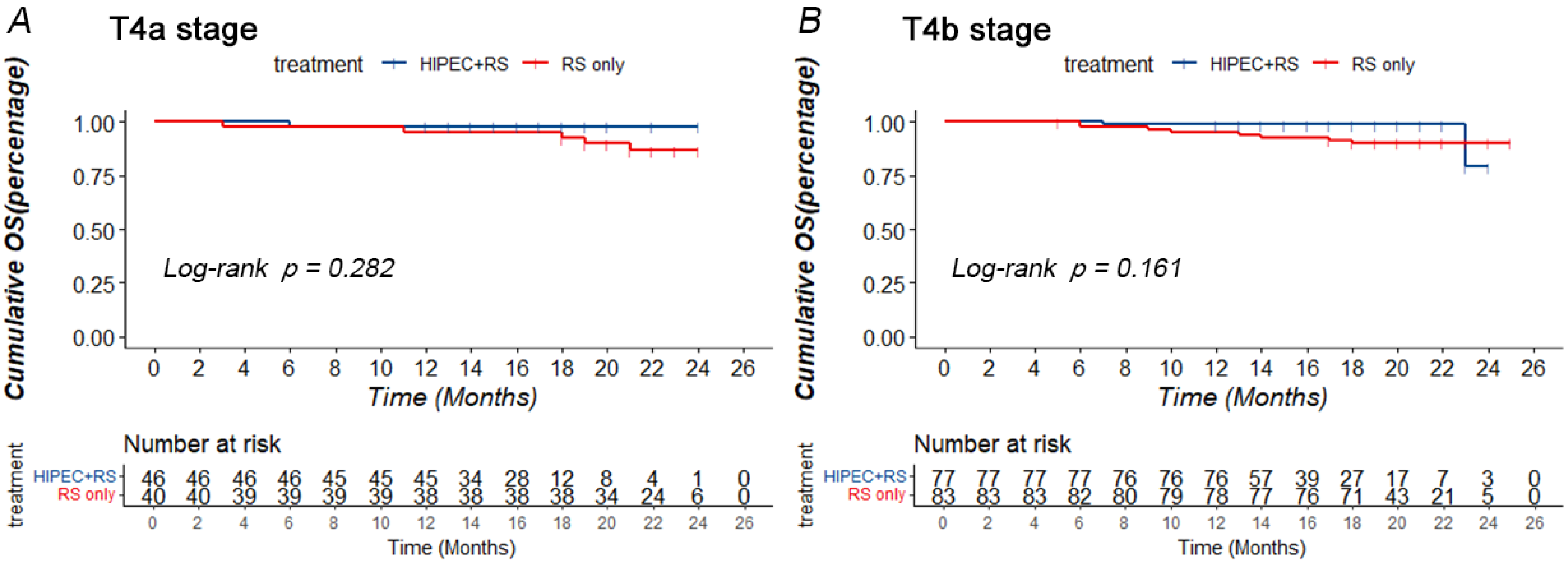

Supplementary Figure S3. Subgroup analysis based on T stage. (A) OS of RS+HIPEC group and RS group after PSM when the tumor at T4a stage. (B) OS of RS+HIPEC group and RS group after PSM when the tumor at T4b stage.

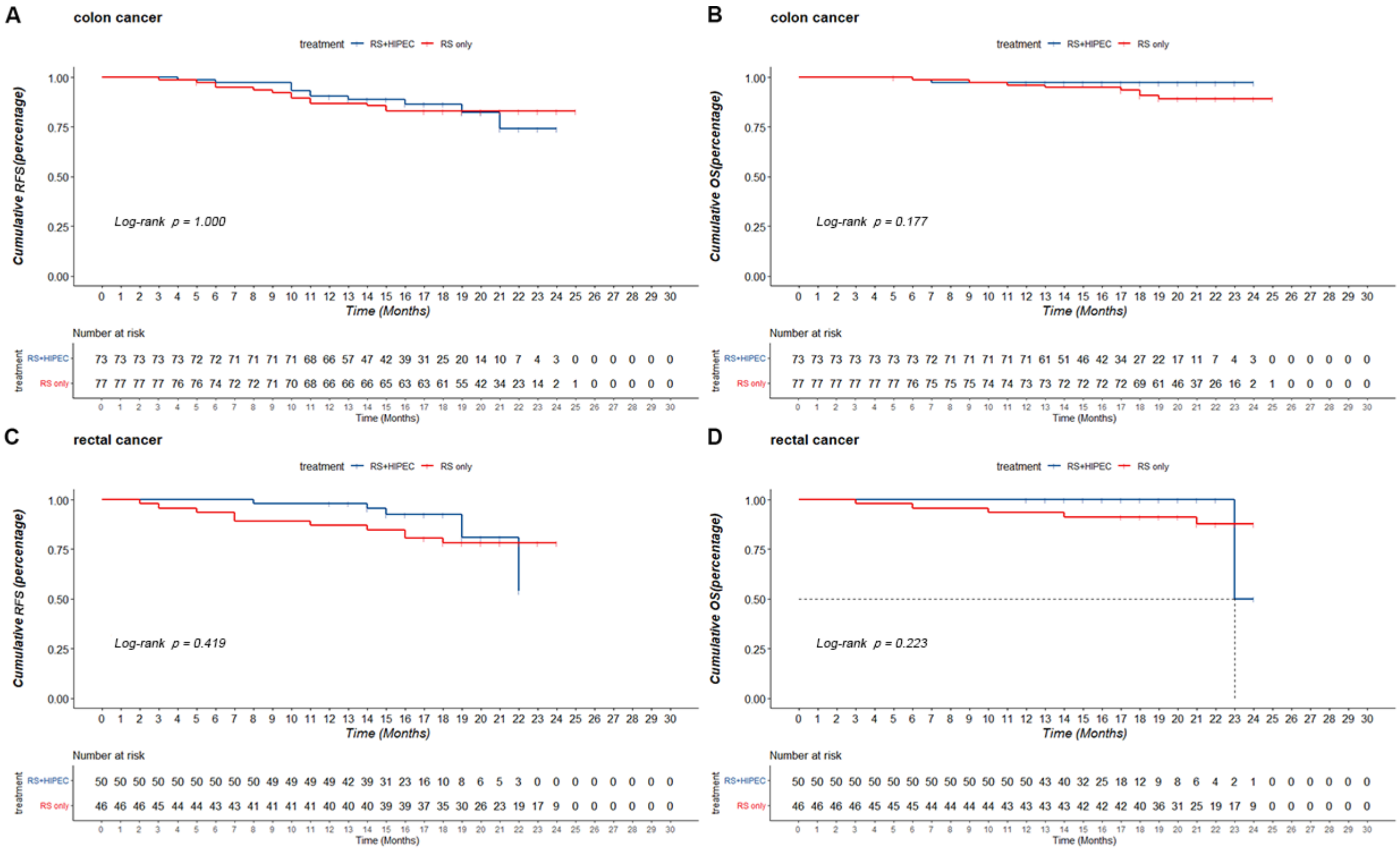

Supplementary Figure S4. Subgroup analysis based on tumor location. (A) RFS of RS+HIPEC group and RS group after PSM when the tumor at colon. (B) OS of RS+HIPEC group and RS group after PSM when the tumor at colon. (C) RFS of RS+HIPEC group and RS group after PSM when the tumor at rectum. (D) OS of RS+HIPEC group and RS group after PSM when the tumor at rectum.

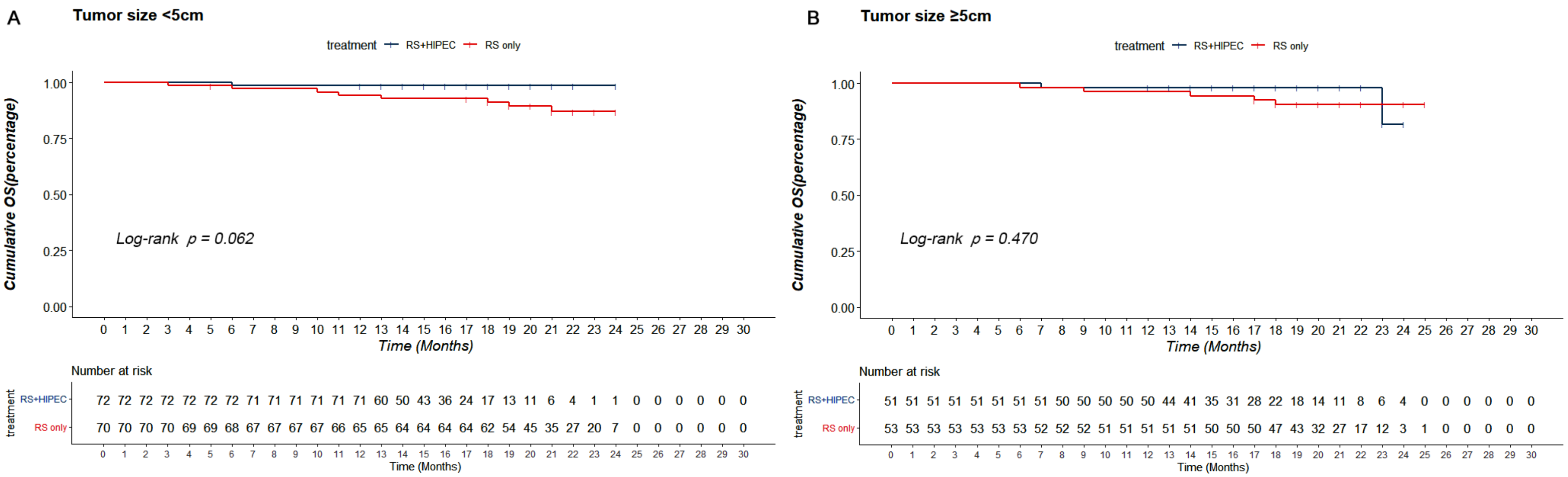

Supplementary Figure S5. Subgroup analysis based on tumor size. (A) OS of RS+HIPEC group and RS group after PSM when the tumor <5cm. (B) OS of RS+HIPEC group and RS group after PSM when the tumor ≥5cm.
